# An Investigation into Spent Coffee Waste as a Renewable Source of Bioactive Compounds and Industrially Important Sugars

**DOI:** 10.3390/bioengineering3040033

**Published:** 2016-11-21

**Authors:** Damhan S. Scully, Amit K. Jaiswal, Nissreen Abu-Ghannam

**Affiliations:** School of Food Science and Environmental Health, College of Sciences and Health, Dublin Institute of Technology, Cathal Brugha Street, Dublin 1, Ireland; damhanscully@gmail.com (D.S.S.); amit.jaiswal@dit.ie (A.K.J.)

**Keywords:** spent coffee waste, lignocellulose, enzymatic saccharification, reducing sugars, polyphenols

## Abstract

Conventional coffee brewing techniques generate vast quantities of spent espresso grounds (SEGs) rich in lignocellulose and valuable bioactives. These bioactive compounds can be exploited as a nutraceutical or used in a range of food products, while breakdown of lignocellulose generates metabolizable sugars that can be used for the production of various high-value products such as biofuels, amino acids and enzymes. Response surface methodology (RSM) was used to optimize the enzymatic saccharification of lignocellulose in SEGs following a hydrothermal pretreatment. A maximum reducing sugar yield was obtained at the following optimized hydrolysis conditions: 4.97 g of pretreated SEGs, 120 h reaction time, and 1246 and 250 µL of cellulase and hemicellulase, respectively. Industrially important sugars (glucose, galactose and mannose) were identified as the principal hydrolysis products under the studied conditions. Total flavonoids (*p =* 0.0002), total polyphenols (*p =* 0.03) and DPPH free-radical scavenging activity (*p =* 0.004) increased significantly after processing. A 14-fold increase in caffeine levels was also observed. This study provides insight into SEGs as a promising source of industrially important sugars and polyphenols.

## 1. Introduction

Coffee is one of the most popular beverages in the world. The International Coffee Organization (ICO) estimates that 8.5 billion kg of coffee was produced in 2014, the majority of which was consumed in the EU, the USA, Brazil and Japan [1]. Due to extensive post-harvest processing of the coffee cherry and conventional brewing techniques, vast quantities of co-products are generated in both producing and consuming regions [2]. Legislation increasingly limits the amount of agri-food waste that can enter landfills; therefore, the generation of waste and co-products represents a significant challenge to the coffee industry [3].

The primary by-product from coffee production is spent coffee grounds (SCGs) [2]. SCGs are the insoluble residue that remains after coffee beans are dehydrated, milled and brewed, and there are two sources: those generated by the soluble coffee industry, which utilizes ~50% of the global coffee harvest each year, and those generated by cafés and the public, accounting for the remaining 50% [4]. Previously, soluble or “instant” coffee producers dumped large quantities of SCGs in landfills, a practice that might alter the local ecology. Incorporating SCGs into animal feed is also limited due to the anti-nutritional activity of tannins [4,5].

SCGs are rich in lignocellulose, a covalently bonded network of lignin, cellulose and hemicellulosic polysaccharides that gives structural stability to the plant cell wall [6]. The structure of lignocellulose is such that covalently cross-linked lignin and hemicellulose sheath a crystalline cellulose core resulting in a strong, recalcitrant network. Consequently, intensive treatment(s) of biomass is required to overcome the covalent linkages between the lignin and hemicellulose [7]. Disrupting lignocellulose in this way improves hydrolysis of hemicellulose and cellulose into industrially significant monosaccharides. Generally, enzymatic saccharification of pretreated lignocellulose is favored over other processing methods as it is environmentally friendly, requires mild reaction conditions, is substrate specific, and produces fewer degradation by-products [8,9]. Nevertheless, efficient enzymatic saccharification of pretreated lignocellulose depends on optimizing experimental variables [10].

Response surface methodology (RSM) is a collection of mathematical and statistical techniques for optimizing and analyzing the effect of multiple experimental variables, alone or in combination, on a given process while maintaining a high degree of statistical significance [11]. RSM optimization is useful because it not only expedites the experimental process, taking into account complex interactions between the variables, but it also reduces operation costs [12]. For these reasons, RSM is routinely used when optimizing enzymatic saccharification of lignocellulosic biomass [10,11,12,13].

To date, the majority of research into coffee by-products has focused on SCGs. SCGs are generated through industrial-scale brewing of coffee beans to produce instant or soluble coffee grounds. Relatively few studies have investigated spent espresso grounds (SEGs), generated through conventional brewing of coffee beans by the foodservice industry (cafés, restaurants) and the public. The present study investigates SEGs as a viable substrate for the production of industrially significant sugars and polyphenols.

## 2. Materials and Methods 

### 2.1. Chemicals and Reagents

The following chemicals and reagents were purchased from Sigma Aldrich, Germany: Hemicellulase from *A. niger*, 2,2-Diphenyl-1-picrylhydrazyl (DPPH); 6-Hydroxy-2,5,7,8-tetramethylchroman-2-carboxylic acid (Trolox), and Multi-element Standard Solution 1 for ICP-AES. Celluclast 1.5L (700 U·mL^−1^) was purchased from Novozymes (Copenhagen, Denmark). All other chemicals were of analytical grade.

### 2.2. Sample Acquisition and Preparation

SEGs were acquired from a local café in Dublin city centre and subsequently dehydrated (Rational Combi-Dämpfer CCC 101, Rational, Landsberg, Germany) at 80 ± 1 °C for 48 h. Dehydrated SEGs were manually sieved to a particle size of 500 µm (Woven Wire Sieve, Endecotts Ltd., London, UK) before being stored at −20 °C until required.

### 2.3. Proximate Composition

#### 2.3.1. Moisture Content

Moisture content was determined as per AOAC method 930.94 [14]. SEGs (5 g) were dehydrated in an oven (Gallenhamp BS Oven 250, Weiss Gallenkamp, Loughborough, UK) for 48 h at 80 ± 2 °C. Weight difference was calculated before and after dehydration to determine the moisture content using Equation (1):

(1) $$\mathrm{Moisture}\;\mathrm{content}\;\left(\%\right)=\left(\frac{\mathrm{Wet}\;\mathrm{sample}\;-\;\mathrm{Dry}\;\mathrm{sample}}{\mathrm{Wet}\;\mathrm{sample}}\right)\times 100$$

#### 2.3.2. pH

pH was determined as per the method of Cruz et al. [4], with minor modifications. SEGs (5 g) were boiled in 50 mL distilled water (dH_2_O) for 5 min with continuous stirring (Bibby HB502, Bibby-Scientific, Staffordshire, UK). The mixture was centrifuged (Sigma 2-16 K, Sigma Zentrifugen, Osterode am Harz, Germany) at 7000 rpm for 10 min. The supernatant was decanted, allowed to cool, then made up to 100 mL with dH_2_O. The pH was measured (Thermo Scientific Orion 2 Star, Thermo Fisher Scientific, Waltham, MA, USA) following calibration with pH 4 and 7 buffer solutions.

#### 2.3.3. Ash Content

Ash content was determined as per AOAC method 920.93 [14]. SEGs (0.5 g) were added to a crucible, previously washed and dried with deionized water, and dry ashed at 500 ± 10 °C in a muffle furnace (Carbolite AAF 11-3, Carbolite, Derbyshire, UK) for 24 h.

#### 2.3.4. Minerals and Trace Elements

Sample preparation for ICP-AES analysis was performed as per the aqua regia method of Szymczycha-Madeja, et al. [15]. Briefly, SEGs (0.5 g) and freshly prepared aqua regia (2 mL) were added to a 30 mL polypropylene centrifuge tube. The mixture was ultrasonicated (Branson 3510 Ultrasonicator, Emerson Electronics, St. Louis, MO, USA) for 15 min and subsequently made up to 25 mL with dH_2_O. Next, the solution was centrifuged at 7000 rpm for 10 min. The supernatant was decanted and stored at −20 °C until required.

ICP (Liberty 150 Series II, Varian, Palo Alto, CA, USA) radio frequency power, plasma flow rate and sheath gas flow rate were 1.1 kW, 10 L·min^−1^ and 0.75 L·min^−1^, respectively. Sample solutions were introduced into the plasma using a V-groove nebulizer and a Scott type spray chamber at a flow rate of 0.7 mL·min^−1^.

#### 2.3.5. Soluble Protein

Hydrolysate protein concentration was determined as per the method of Bradford [16]. Briefly, boiled and filtered SEGs solution (200 µL) was mixed with Bradford reagent (800 µL) and allowed to stand for 5 min at 25 °C. Absorbance was measured (Shimadzu UV-1800, Shimadzu Corp., Kyoto, Japan) at 595 nm.

#### 2.3.6. Total Lipids

Lipids were extracted as per the method of De Melo et al. [17]. SEGs (10 g) were added to a cellulose thimble and subsequently placed in a Soxhlet extraction chamber. Lipids were extracted over 6 h by refluxing in 100 mL *n*-hexane. After 6 h extraction lipids were concentrated by evaporating *n*-hexane (Büchi Rotovapor R-215, Büchi, Postfach, Switzerland).

### 2.4. Enzymatic Hydrolysis of Spent Espresso Coffee Lignocellulose

#### 2.4.1. Preliminary Study

SEGs (5 g) and dH_2_O (40 mL) were added to an Erlenmeyer flask (100 mL). Each mixture was hydrothermally pretreated (autoclaved) at 120 °C and 15 psi for 1 h and subsequently allowed to cool to 25 °C. Next, 50 mM sodium citrate buffer (5 mL) was added to each flask. A volume (0.5, 1.0, 1.5 or 2.0 mL) of cellulase (77.08 U·mL^−1^) or hemicellulase solution (7.23 U·mL^−1^) was then added to the flasks. The final volume was made up to 50 mL with dH_2_O. The flasks were incubated at 40 °C for 24, 48, 72, 96 and 120 h, respectively. After incubation, each suspension was centrifuged at 7000 rpm for 10 min. Hydrolysates were stored at −20 °C until required. Table 1 shows the range of values established from the preliminary study.

#### 2.4.2. Central Composite Design

A 2^4^ factorial Box-Wilson Central Composite Design (CCD) was used for experimental design. A total of 28 experiments were generated by the software to determine the optimum levels of the process variables SEGs quantity (Χ_1_), cellulase volume (Χ_2_), hemicellulase volume (Χ_3_) and reaction time (Χ_4_). All variables were assumed to be measurable; therefore, the response surface was expressed using Equation (2):

(2) $$y=f({Χ}_{1},{Χ}_{2},{Χ}_{3},{Χ}_{4})$$

where *y* is the response variable and Χ*_i_* (*i* = 1, 2, 3, 4) represents the process variables.

To find the values that optimally describe the functional relationship between the process variables and the response surface a second-order polynomial model (Equation (3)) was utilized:

(3) $$y=\beta_{0}+\overset{k}{\underset{i=1}{\sum }}\beta_{i}{Χ}_{i}+\overset{k}{\underset{i=1}{\sum }}\beta_{ii}{Χ}_{i}^{2}+\overset{k-1}{\underset{i=1}{\sum }}\overset{k}{\underset{j=2}{\sum }}\beta_{ij}{Χ}_{i}{Χ}_{j}+\varepsilon$$

where Χ_1_,…Χ*_k_* are the process variables that influence the response *y*; β_0_, β*_ii_* (i = 1, 2,...k), β*_ij_* (*i* = 1, 2,...k; *j* = 1, 2,...k) are the unknown parameters of the variables; and ε the random error.

### 2.5. Analysis of Reducing Sugars

#### 2.5.1. The DNS Assay

The dinitrosalicylic acid (DNS) assay was used to quantify total reducing sugars in the preliminary study and RSM-optimized hydrolysates. Briefly, each hydrolysate (1 mL) was diluted 1:10 in dH_2_O before addition of dinitrosalicylic acid reagent (1 mL) and dH_2_O (2 mL). The mixture was submerged in a water bath for 5 min at 100 °C. The volume was made up to 10 mL by the addition of dH_2_O (6 mL). Absorbance was measured at 540 nm.

#### 2.5.2. HPLC-RI

Reducing sugars were analyzed using the method described by Jaiswal et al. [18]. An Alliance High Performance Liquid Chromatography (HPLC) (Waters, e2695 Separation module, Waters, Milford, MA, USA) equipped with an auto-sampler and controller with dual pump was used. The detection system consisted of a Waters 486 UV detector and a Waters 410 Differential Refractometer (RI detector) connected in series. Empower^®^ software was used for data acquisition and analysis. An aliquot (20 µL) was injected into a thermostatically controlled compartment set to 65 °C, followed by elution through a Rezex ROA-Organic acid H+ (8%) (350 mm × 7.8 mm, Phenomenex, Macclesfield, UK) column fitted with a guard column (50 mm × 7.8 mm, Phenomenex, Macclesfield, UK), at a flow rate of 0.6 mL·min^−1^. The mobile phase was H_2_SO_4_ (5 mM). All solutions were filtered through a 0.22 µm Millipore^®^ filter (Millipore, Merck-Millipore, Billerica, MA, USA) before being injected into the instrument.

### 2.6. Analysis of Caffeine, Polyphenols and Antioxidant Activities

#### 2.6.1. Total Flavonoids Content (TFC) Assay

Total flavonoids content (TFC) was measured using the method described by Rajauria et al. [19]. Briefly, an aliquot (250 µL) was mixed with dH_2_O (1.25 mL) and 5% NaNO_2_ (75 µL ). After 6 min, 10% AlCl_3_ (150 µL) was added. After 5 min, 0.1 M NaOH (0.5 mL) was added. The total volume was made up to 2.5 mL with dH_2_O. Absorbance was measured at 510 nm. Results are expressed as quercetin equivalents (QE).

#### 2.6.2. Total Polyphenol Content (TPC) Assay

Total polyphenol content (TPC) was measured using the FC method described by Rajauria et al. [18]. An aliquot (100 µL) was mixed with 2% Na_2_CO_3_ (2 mL) and allowed to stand for 2 min at 25 °C. Next, 1 N FC reagent (100 µL) was added. The sample was incubated in darkness for 30 min at 25 °C. Absorbance was measured at 720 nm. Results are expressed as gallic acid equivalents (GAE).

#### 2.6.3. DPPH Assay

The DPPH assay was used to determine the free-radical scavenging activity (EC_50_) of preliminary study hydrolysates, but not in the RSM-optimized hydrolysates due to the inclusion of citric acid as a buffer [20]. Briefly, aliquots (100 µL) were added to a 96-well plate followed by the addition of 0.16 mM methanolic DPPH solution (100 µL). The mixture was shaken and incubated in darkness for 30 min at 25 °C. Absorbance was monitored at 517 nm (Powerwave, Biotek, Winooski, VT, USA). The ability to scavenge the DPPH radical was calculated using Equation (4):

(4) $$\mathrm{Scavenging}\;\mathrm{effect}\;\left(\%\right)=\left[1-\left(\frac{A_{s}-A_{b}}{A_{c}}\right)\right]\times 100$$

where *A_s_* is the absorbance of the test sample; *A_b_* is the absorbance of the sample blank; and *A_c_* is the absorbance of the control. Results are expressed as ascorbic acid equivalents (AAE).

#### 2.6.4. FRAP Assay

The ferric reducing antioxidant potential (FRAP) of preliminary study hydrolysates was assessed using the method of Benzie and Strain [21]. The assay was performed in a 96-well microtitre plate reader at 37 °C. An aliquot of FRAP reagent (100 µL) was preheated to 37 °C and subsequently dispensed in wells containing hydrolysates (50 µL). Absorbance was measured at 593 nm after 10 min. Results are expressed as trolox equivalents (TE).

#### 2.6.5. Analysis of Caffeine Using HPLC-DAD

Caffeine was identified as per the method of Jaiswal et al. [18]. The system consisted of a reversed-phase column in an Alliance HPLC equipped with an auto-sampler and controller with dual pump, a Waters 2998 photodiode array detector (DAD) set to 280 nm, and the Empower^®^ software. An Atlantis C_18_ column (250 mm × 4.6 mm, 5 μm particle size) maintained at 25 °C was used. A gradient solvent system of 2 mM sodium acetate with 6% acetic acid (Solvent A) and acetonitrile (Solvent B) gave optimal peak separation and resolution.

### 2.7. Statistical Analysis

All samples were prepared in triplicate and results are reported as the mean ± the standard deviation. Preliminary and RSM optimization experiments were conducted in triplicate. Experiment design and statistical and regression analyses were carried out in STATGRAPHICS Centurion XV^®^ software (StatPoint Technologies, Warrenton, VA, USA). Lack-of-fit, the *R*^2^ statistic and analysis of variance (ANOVA) *p*-values were analyzed to determine if the model was adequate.

## 3. Results and Discussions

### 3.1. Proximate Analysis of SEGs

Two major issues arise when agri-food by-products are not sufficiently dehydrated: (i) contamination of the feedstock by spoilage microorganisms resulting in fermentation with concomitant acidification; and (ii) decreased efficiency of the pretreatment [8]. In both instances the hydrolytic activity of enzymes can be affected. Therefore, dehydrating biomass to a moisture content of ≤10% is recommended [22]. The moisture content of SEGs used in this study was 65.3% ± 0.4%. This compared favorably with values for SEGs (53.0%–69.8%) previously reported by Cruz et al. [4].

The ash content of roasted coffee is ~4.6%; however, conventional brewing can reduce this by as much as 93%, owing to the hydrophilic nature of inorganic compounds and minerals [23,24]. The ash content of SEGs used in our study was 0.7% ± 0.1%. This was lower than the range (0.8%–3.5%) reported by Cruz et al. [4]. Mussatto et al. [24] conducted a similar analysis on SCGs and found a lower ash content, suggesting that brewing conditions used in industrial coffee extraction result in greater loss of ash and minerals. Examining the ash in greater detail, we identified and quantified eight elements in SEGs, namely potassium, magnesium, calcium, manganese, copper, sodium, iron and zinc (Table 2). Phosphorus was not included in our analysis; however, this element is present at high concentration in both SCGs and SEGs [4,24]. The concentrations of potassium, magnesium and calcium followed the same trend (potassium > magnesium > calcium) as values reported by Cruz et al. [4] and Mussatto et al. [24], despite large variations between the values. The minor elements—manganese, copper, sodium, iron and zinc—were lower than those previously reported.

The concentration of acids in the coffee bean is largely influenced by species, growth conditions, processing method(s), degree of roasting and brewing method. The unroasted green coffee bean is comprised of ~11% acids (w·w^−1^), including phenolic acids such as chlorogenic acid; non-volatile aliphatic acids, such as citric and malic acids; and volatile acids such as acetic and propanoic acids, all of which contribute to coffee’s complex flavor and aroma profile [4]. Roasting effectively reduces coffee bean acidity to ~6% (w·w^−1^) and, once brewed, Arabica varieties typically are between pH 5.02 and 5.45, whereas Robusta varieties are between pH 5.32 and 5.49 [23,25]. The pH of brewed coffee is an important indicator of quality; however, as mentioned above, low pH can reduce enzyme activity and other saccharification and fermentation bioprocesses. The pH of a secondary extraction was 5.1 ± 0.0. Compared with pH values reported for SCGs (pH 4.9 ± 0.9) by Kostenberg and Marchaim [26], a secondary extraction had a negligible effect on brew acidity when compared with industrial processes.

Despite comprising a modest percentage of the coffee bean (11%–20% w·w^−1^), lipids are a minor constituent in the beverage because they are poorly extracted by hot water, making SEGs a good source. Arabica beans have higher levels than Robusta (14%–20% vs. 11%–16%), with the principal lipids being linoleic acid (~40%); palmitic acid (~30%); oleic acid (~10%); and stearic acid (~7%), and coffee bean lipids contribute to the sensory profile of coffee [4,27]. Our analysis revealed that SEGs were comprised of 13.1% ± 0.8% (dry w·w^−1^), making them a good source of lipids.

### 3.2. Optimization of Variables to Maximize Lignocellulose Saccharification

Following the preliminary study, a 24-factorial Box-Wilson CCD was used to design experiments. A total of 28 experiments were generated by the software to determine the optimum levels of the process variables SEGs (g), cellulase volume (µL), hemicellulase volume (µL) and reaction time (h) (Table 3). A multiple regression analysis of the observed reducing sugar values (Table 3) revealed that the variables were related by a second-order polynomial equation (Equation (5)):

(5) $$y=-2.4775+2.80458{Χ}_{1}+0.0023{Χ}_{2}+0.00072{Χ}_{3}+0.042{Χ}_{4}-0.150{Χ}_{1}^{2}+0.00082{Χ}_{1}{Χ}_{2}+0.00001{Χ}_{1}{Χ}_{3}+0.029{Χ}_{1}{Χ}_{4}+0.0000045{Χ}_{2}^{2}-0.0000035{Χ}_{2}{Χ}_{3}-0.000057{Χ}_{2}{Χ}_{4}-0.0000064{Χ}_{3}^{2}-0.000063{Χ}_{3}{Χ}_{4}-0.00095{Χ}_{4}^{2}$$

where${\;Χ}_{1},{\;Χ}_{2},{\;Χ}_{3}\;$and${\;Χ}_{4}\;$are coded values for SEGs (g), cellulase volume (µL), hemicellulase volume (µL) and reaction time (h), respectively. 

Analysis of variance (ANOVA) was carried out and the results show that the SEGs’ quantity (*p =* 0.000), reaction time (*p =* 0.000) and cellulase volume (*p =* 0.003) were significant variables with *p =* 0.05. The effect of hemicellulase was not significant (*p =* 0.228). This is likely due to the activity of this particular enzyme. For example, Jooste et al. [28] reported that mannanase gave the optimal hydrolysis of spent coffee hemicellulose as compared with xylanase or cellulase. The significance of the regression equation was checked by the F-test, and the *R*^2^ statistic showed that the fitted model explained 94.897% of the variability between observed and predicted values. The adjusted *R*^2^ statistic was 89.402%, meaning that the models could predict most of the observed variability. The standard error of the estimates showed that the prediction errors of the residuals were 1.7069 (mg·g^−1^), while the absolute mean error of the residuals was 0.9025 (mg·g^−1^). Finally, the Durbin-Watson statistic was used to test if there was a significant correlation based on the order in which the residuals occurred. This was 1.8821 (mg·g^−1^), or *p =* 0.235. Since *p* > 0.05, there was no indication of serial auto-correlation in the residuals.

**Figure 1:**
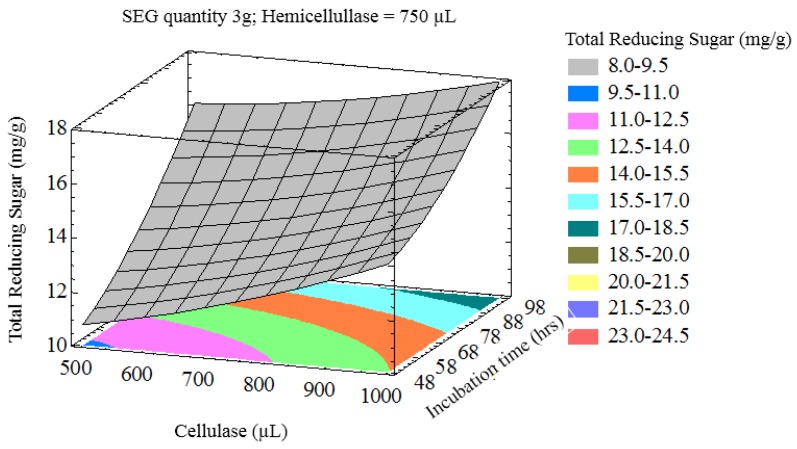
Three-dimensional response surface plot showing influence of cellulase (uL) and incubation time (h) when the response surface is fixed at a spent espresso grounds (SEG) quantity = 3 g and hemicellulase = 750 uL.

**Figure 2:**
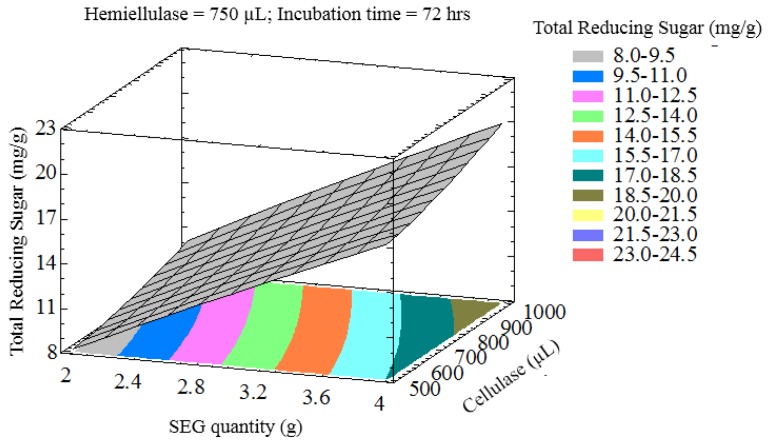
Three-dimensional response surface plot showing influence of SEG quantity and cellulase loading when the response surface is fixed at an incubation time = 72 h and hemicellulase = 750 uL.

**Figure 3:**
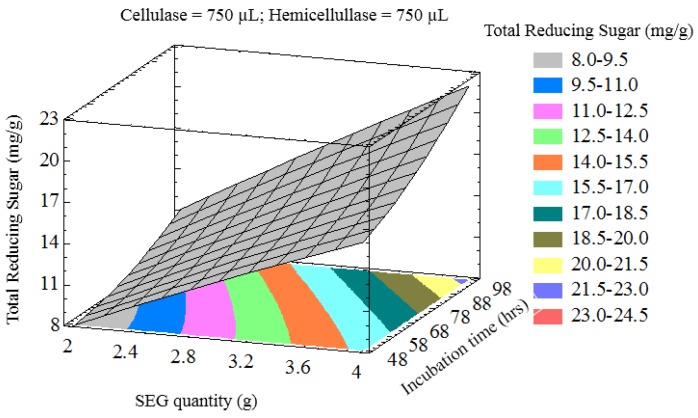
Three-dimensional response surface plot showing influence of SEG quantity (g) and incubation time (h) when the response surface is fixed at cellulase and hemicellulose loadings = 750 uL.

**Figure 4:**
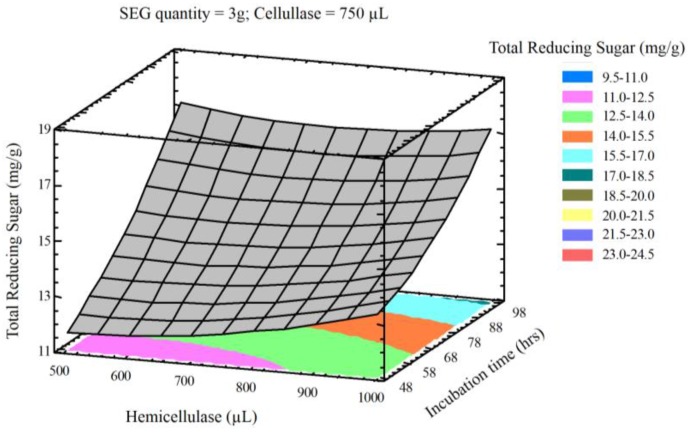
Three-dimensional response surface plot showing influence of incubation time (h) and hemicellulase (uL) loading when the response surface is fixed at SEG quantity (3 g) and cellulase = 750 uL.

The interaction of process variables is depicted in selected three-dimensional response surface plots (Figure 1, Figure 2, Figure 3 and Figure 4). As shown in Figure 1, the cellulase volume and time produced a non-linear response surface when the SEG quantity (3 g) and hemicellulase volume (750 µL) were fixed. The reaction time had a large effect on the reaction under these conditions, while increasing the cellulase volume also increased the reducing sugar yield. The synergy between SEGs and cellulase volume when the reaction time and hemicellulase volume were 72 h and 750 µL is shown in Figure 2. A concomitant increase in SEGs and cellulase volume effectively doubled the concentration of reducing sugars. Under the conditions shown in Figure 3 and Figure 4, as time increased from 48 to 98 h the reducing sugar yield increased linearly. In Figure 4, however, it is clear that under optimized conditions the hemicellulase volume has a negligible response on the reducing sugar concentration.

### 3.3. Optimization of Reducing Sugar Yield

The aim of optimizing process variables was to find the combination of values that would maximize reducing sugar yield. Optimal values were obtained by solving the regression equation (Equation (5)) using STATGRAPHICS Centurion XV^®^ software (STATGraphics Centurion, Warrenton, VA, USA). Table 4 shows the values required to maximize reducing sugar yield. SEGs and cellulase volume were rounded to 5 g and 1250 µL, respectively. Based on the optimum values, the predicted maximum reducing sugar yield was 379.0 mg·g^−1^. The observed maximum reducing sugar yield was 325.6 mg·g^−1^. Our results show that optimizing the process variables effectively increased the total reducing sugars. For example, reducing sugars in the RSM-optimized hydrolysate were 4.4 times more concentrated than the equivalent pretreated hydrolysate (50 mg·g^−1^) and 11.7 times more concentrated than the control (18.5 mg·g^−1^).

### 3.4. Identification of Reducing Sugars

**Figure 5:**
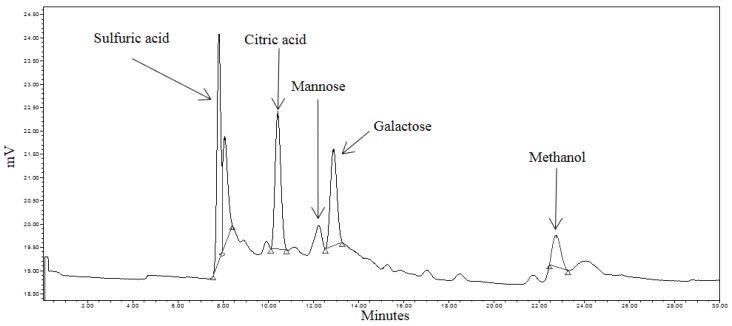
HPLC (High Performance Liquid Chromatography) chromatogram of spent coffee waste (control) sample.

**Figure 6:**
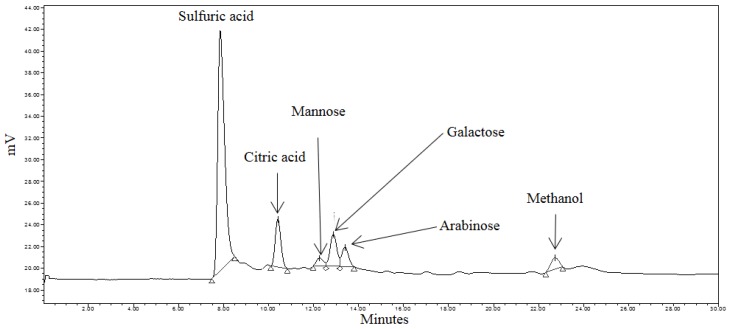
HPLC chromatogram of pretreated spent coffee waste sample.

**Figure 7:**
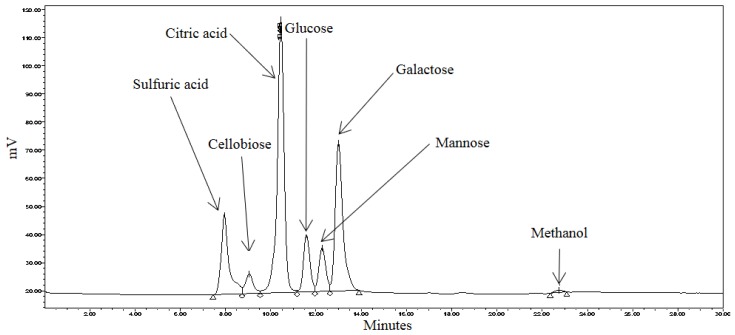
HPLC chromatogram of pretreated spent coffee waste sample.

Liberated sugars in the control, pretreated and RSM-optimized hydrolysates are shown in Figure 5, Figure 6 and Figure 7. Peaks at approximately 8.0 min, 10.5 min, and 22.7 min in all chromatograms represent H_2_SO_4_, citric acid, and MeOH, respectively. Sodium citrate was used as a buffer in all the sample solutions, 0.005 M H_2_SO_4_ was used as the mobile phase throughout all analyses, and MeOH was used to equilibrate the column prior to running the hydrolysates. Most six-carbon sugars eluted around 12 min due to structural similarities. As shown in Figure 5, only mannose and galactose were identified in the control.

The effects of hydrothermal pretreatment conditions on SEGs were evident as mannose, galactose and arabinose were identified in the pretreated hydrolysate (Figure 6). It was expected that these sugars would be identified during the course of the analysis as they comprise the hemicellulosic arabinogalactans and galactomannans in spent coffee [29]. The concentrations of mannose and arabinose in the pretreated hydrolysate were 0.087 mg·mL^−1^ and 0.108 mg·mL^−1^, respectively.

As shown in Figure 7, the variety of sugars was significantly higher in the RSM-optimized hydrolysate. Glucose, mannose and galactose were present, in addition to cellobiose. The presence of cellobiose and glucose was indicative of cellulase activity. The hydrothermal pretreatment effectively disrupted the SEGs’ lignocellulosic structure to the extent that cellulase could effectively degrade cellulose. Interestingly, arabinose was not identified in the RSM-optimized hydrolysate. Retention time data (not shown) supports the explanation that arabinose co-eluted with galactose and that its peak was engulfed by the galactose peak. The concentrations of cellobiose, glucose and mannose in the RSM-optimized hydrolysate were 3.258 mg·mL^−1^, 6.501 mg·mL^−1^ and 5.660 mg·mL^−1^, respectively. The concentrations of cellobiose and glucose were high considering that these sugars were undetectable in the control and SA hydrolysates. The concentration of mannose was 65 times greater in the RSM-optimized hydrolysate when compared with the SA hydrolysate.

### 3.5. Caffeine, Polyphenols and Antioxidant Activities

Hydrothermal pretreatment of SEGs increased the concentration of flavonoids and phenolic acids in the hydrolysates. For example, when compared with the control, pretreatment increased flavonoids by 24.0% (495.8 ± 0.2 µg·QE·g^−1^ vs. 651.4 ± 1.1 µg·QE·g^−1^), while phenolics increased by 33.9% (417.6 ± 0.3 µg·GAE·g^−1^ and 631.4 ± 0.8 µg·GAE·g^−1^). Student’s *t*-test was used to determine if a significant difference existed between the concentration of flavonoids and phenolics in the control and pretreated hydrolysate. The increase in flavonoids (*p =* 0.03) and phenolics (*p =* 0.00002) resulting from pretreatment was significant when *p =* 0.05. Pretreatment effectively disrupted complexes between phenolic compounds and lignin, polysaccharides and proteins. When compared with values for unbrewed coffee, the data also highlighted the efficacy of conventional brewing on polyphenols extraction. For example, Cheong et al. [30] found total phenolics in roasted coffee to be 33.67–43.13 mg GAE·g^−1^, while Hečimović et al. [31] reported the total flavanoid content of coffee as 17.29 mg·GAE·g^−1^–20.58 mg·GAE·g^−1^. 

Antioxidant activities of the control and pretreated hydrolysate were determined by measuring the DPPH free-radical scavenging activity and FRAP. The DPPH free-radical scavenging activity (EC_50_) was greater for the pretreated hydrolysate (59.9%) than the control (47.4%). FRAP results showed that there was no significant difference between pretreated hydrolysate (82.8 ± 2.1 μg·TE·g^−1^) and the control (82.0 ± 2.1 μg·TE·g^−1^). Student’s *t*-test was used to determine if a significant difference existed between the DPPH free-radical scavenging activities and FRAP values of the control and pretreated hydrolysate. When *p =* 0.05, there was no significant difference between the FRAP values (*p =* 0.403); however, differences between the DPPH values were significant (*p =* 0.004). One explanation for this is the difference between the electron transfer mechanism(s) in the assays. For example, the FRAP assay measures antioxidants capable only of a single electron transfer, whereas the DPPH assay measures antioxidants that transfer both a single electron and a hydrogen atom. Hence, obtaining a good agreement between the assays is difficult [32].

We identified and quantified caffeine in the control, pretreated and RSM-optimized hydrolysates. Our analysis revealed that pretreatment resulted in a 14-fold increase in caffeine when compared with the control (110.8 µg·mL^−1^ vs. 7.8 µg·mL^−1^). When compared against the pretreated hydrolysate, however, enzymatic saccharification only slightly increased the caffeine concentration (118.2 µg·mL^−1^).

## 4. Conclusions

This study highlighted SEGs, an underutilized source of biomass, as a promising source of industrially important reducing sugars and extractable bioactives, while also revealing the importance of optimizing experimental parameters and selecting appropriate enzyme(s) when hydrolyzing or processing lignocellulosic biomass. A deeper analysis and more in-depth studies of SEGs and the process chain are required. SEGs can be systematically exploited as biomass for the production of fermentable sugars glucose, mannose, galactose and arabinose, in addition to extractable lipids, polyphenols and caffeine.

## Figures and Tables

**Table 1:** Process variables and their coded levels in the Box-Wilson Central Composite Design.

Process Variables	Coded Values				
	−2	−1	0	1	2
Χ1: SEG Quantity (g)	1	2	3	4	5
Χ2: Cellulase (µL)	250	500	750	1000	1250
Χ3: Hemicellulase (µL)	250	500	750	1000	1250
Χ4: Incubation Time (h)	24	48	72	36	120

**Table 2:** Trace elements in spent coffee grounds

Trace Elements	Concentration (mg·(100 g)^−1^)
Potassium	258.2 ± 23.66
Phosphorus	ND
Magnesium	49.6 ± 3.34
Calcium	37.2 ± 2.89
Manganese	1.8 ± 0.06
Copper	1.2 ± 0.19
Sodium	1.1 ± 0.01
Iron	0.9 ± 0.02
Zinc	0.1 ± 0.03

ND = Not Determined.

**Table 3:** Experiment conditions as determined by STATGraphic Centurion XV software^®^ (STATGraphics Centurion, Warrenton, VA, USA). Also included are predicted and observed reducing sugar values for each condition.

Experiment	Process Variables *				Concentration (mg·mL^−1^)	
	Χ_1_ (g)	Χ_2_ (µL)	Χ_3_ (µL)	Χ_4_ (h)	Predicted	Observed
1	3	750	750	120	20.38	18.37
2	3	750	750	72	13.76	12.76
3	3	250	750	72	12.35	10.94
4	4	1000	500	96	23.69	26.05
5	4	1000	500	48	17.77	17.79
6	5	750	750	72	22.44	19.65
7	4	500	500	48	13.70	14.57
8	4	1000	1000	48	18.97	20.80
9	3	750	750	72	13.76	14.12
10	2	500	1000	48	8.32	7.76
11	4	1000	1000	96	23.38	23.24
12	4	500	1000	96	21.56	23.20
13	2	500	1000	96	11.27	12.14
14	3	1250	750	72	17.42	16.14
15	2	500	500	96	10.71	10.69
16	2	500	500	48	6.24	7.27
17	1	750	750	72	3.88	3.97
18	4	500	1000	72	15.79	16.55
19	3	750	750	96	13.76	13.91
20	3	750	250	72	14.48	13.09
21	2	1000	500	96	12.59	12.72
22	3	750	750	72	13.76	14.24
23	2	1000	1000	96	12.27	13.21
24	3	750	1250	72	16.25	14.94
25	3	750	750	24	11.51	10.82
26	2	1000	500	48	9.50	9.66
27	2	1000	1000	48	10.69	10.65
28	4	500	500	96	20.99	21.92

* Χ_1_: Spent espresso grounds (SEG) quantity; Χ_2_: Cellulase; Χ_3_: Hemicellulase; Χ_4_: Time.

**Table 4:** Low, high, and optimum values for the process variables used in this study.

Process Variables	Limit Values		Optimum Values
	Low	High	
SEG Quantity (g)	1	5	4.97
Cellulase (µL)	250	1250	1246.07
Hemicellulase (µL)	250	1250	250
Incubation Time (h)	24	120	120
